# Religious competence as cultural competence

**DOI:** 10.1177/1363461512439088

**Published:** 2012-04

**Authors:** Rob Whitley

**Affiliations:** McGill University

**Keywords:** religion, cultural competence, recovery, spirituality, mental health services, African American

## Abstract

Definitions of cultural competence often refer to the need to be aware and attentive to the religious and spiritual needs and orientations of patients. However, the institution of psychiatry maintains an ambivalent attitude to the incorporation of religion and spirituality into psychiatric practice. This is despite the fact that many patients, especially those from underserved and underprivileged minority backgrounds, are devotedly religious and find much solace and support in their religiosity. I use the case of mental health of African Americans as an extended example to support the argument that psychiatric services must become more closely attuned to religious matters. I suggest ways in which this can be achieved. Attention to religion can aid in the development of culturally competent and accessible services, which in turn, may increase engagement and service satisfaction among religious populations.

One dimension of human activity that has long fascinated a small but dedicated cadre of researchers within psychiatry is that of religion and religious practice (Bhugra 1996; Koenig, 2009). This is not surprising, given that before psychiatry emerged as a distinct medical discipline, “mental illness” was often interpreted through a religious, moral, or existential prism (Schumaker, 1992; Weatherhead, 1951). Sociocultural research suggests this is still the case for many cultures and subcultures (Kirmayer, 1989, 2004).

Many of the founding fathers of psychiatry had an active interest in religion. While figures such as Jaspers and Jung attempted to integrate psychiatry and religion (e.g., Jaspers, 1971; Jung, 1960), other influential figures were emphatically antireligious. Freud took a critical stance, opining that religious thinking represented a self-deceiving and stagnant infantilism (which aggregated to a collective delusion) on the part of adults that could be redressed through therapy (Freud 1927/1989). These negative attitudes within psychoanalysis were further entrenched by key 20th-century figures in other schools of psychology such as B. F. Skinner and Albert Ellis, who were both resolutely antireligious (Nielsen & Ellis, 1994; Skinner, 1971). Opposing perspectives on the role of religion in psychiatry persist to the present, with tensions manifesting themselves during debates at psychiatric conferences, or in the letters pages of psychiatric journals after publication of an article discussing religion and mental health (e.g., Edmondson, 2010; Hansen & Maguire, 2010; Mackin, 2010). However, in recent years there is some evidence of a growing rapprochement (or at least a desire for one) between religion and psychiatry, with the aim of better integration of the two in order to enhance recovery and healing (Cook, 2010; Dein, Cook, Powell, & Eagger, 2010; King & Leavey, 2010). This paper will address this rapprochement and its implications for the notion of religious competence as a component of clinical cultural competence.

## Religiosity and mental health

Studies of the relationship between psychiatry and religion have been an enduring focus of medical anthropology. Many anthropologists continue to investigate the role of religious healing, shamanism, and religious worldviews on mental illness domestically and abroad (Dura-Vila, Dein, Littlewood, & Leavey, 2010; Harvey, 2003; Kirmayer & Valaskakis, 2009; Winkelman, 2010). Additionally, edited volumes and thoughtful reviews on the relationship between religion and psychiatric illness have been produced within the medical literature (e.g., Bhugra, 1996; Koenig, 1998, 2009). These include a 1997 special issue of *New Directions in Mental Health*, a 2007 special issue of the *Psychiatric Rehabilitation Journal*, and a 2010 special issue of *Transcultural Psychiatry*. The growing corpus of empirical work has been subject to a recent systematic review, suggesting a positive association between religiosity and mental health (Koenig, 2009). This is consistent with an earlier systematic review by the same author (Koenig, 1998), as well as other reviews which suggest that religiosity has a moderately salutary effect on the natural history of mental illness, and can also act as a protective factor for those without mental illness (e.g., Galanter, 2005; Levin, 1996). A consistent body of research also indicates an inverse correlation between religiosity and substance abuse (Huguelet, Borras, Gillieron, Brandt, & Mohr, 2009; Longshore, Anglin, & Conner, 2009). Likewise, another recent review indicates a positive association between religiosity and better physical health and well-being in almost all well-controlled studies (Koenig & Cohen, 2002).

These reviews have met with some criticism. It has been argued that many of the studies linking religiosity and mental health are methodologically flawed. Most are cross-sectional in nature, statistical associations are weak, appropriate corrections for multiple tests are not always performed, and some lack an adequate control group or fail to control for confounders such as disability (King & Leavey, 2010; Sloan & Bagiella, 2002; Sloan, Bagiella, & Powell, 1999). Despite these critiques, in assessing the evidence base and the quality of systematic reviews, most disinterested observers have concluded that the evidence suggests a positive (though modest) association between religiosity and mental health (Blazer, 2009).

This raises the question, what is it about religiosity that is salutogenic? It has been argued that the religious worldview (and concomitant communal activities) held by religiously inclined people (regardless of ethno-cultural background) provides what Antonovsky (1987) called a “sense of coherence.” Religion can be an integral part of the narrative shaping of human experience, imparting meaning and an organizing framework to individual and communal experience, which is an invaluable resource in times of adversity, distress, and suffering (Kleinman, 2006). It also provides access to a community of people who can offer material, moral, emotional, and social support (Durkheim, 1912/2001). These frameworks of meaning and community are protective of general mental health, but may be especially important in times of heightened stress and vulnerability.

## Religiosity and minority mental health: The case of African Americans

Religiosity has significantly declined in most western countries in the last 100 years (Taylor, 2007). The one exception to this trend is the United States, where religiosity remains high in most ethno-cultural groups, with Euro–Americans and African Americans among the most churched people in the world (Berger, 1999). While the literature suggests that religiosity may benefit all who possess it, this may be especially amplified for those lacking other resources, for example low-income people of color (Neighbors, Musick, & Williams, 1998). Much work suggests that people of color fare most poorly within most western mental health care systems. Research in the UK, the USA, and Canada suggests that factors such as cost, cultural incompetence, institutionalized racism, victim blaming, and general lack of sensitivity towards minority issues leads to worse outcomes for minorities in these systems (Cantor-Graae & Selten, 2005; Fernando, 2003; Institute of Medicine, 2002; Jarvis, Kirmayer, Jarvis, & Whitley, 2005; Surgeon General, 2001).

Interestingly, people of color generally have very high rates of theism. For example, surveys suggest that over 95% of African Americans believe in a transcendent deity, higher than the already high rates of Euro–Americans (Levin, Taylor, & Chatters, 1995; Neighbours et al., 1998). Likewise common indicators of religiosity are very high in this population, including rates of church going and prayer, which are again higher than the already high rates in the general U.S. population (Taylor, Chatters, & Levin, 2004). Historically, the Black Church in the USA (referred to in earlier times as the Negro Church) has provided immeasurable social, economic, and political support to the African American people (Frazier, 1974; Lincoln, 1974). This includes the provision of formal and informal social welfare, educational and health services to the African American community in the face of ongoing issues of racism, institutional discrimination, segregation, and socioeconomic disparity. The existence, legitimacy, credibility, and impact of these religiously based institutions, what has been termed a “nation within a nation” is often unrecognized by wider Euro–American society (Frazier, 1974; Lincoln, 1974). The Black Church is one of the few minority institutions in the USA that transcend time and space throughout pre- and postemancipation America, nurturing almost all of the key recent African American leaders including Rev. Dr. Martin Luther King, Rev. Jesse Jackson, Rev. Al Sharpton, and President Barack Obama (Obama, 1995).

As such, the Black Church is an enduring institution that became an essential vehicle for Black emancipation and intracommunity cohesion. The Black Church and African American religiosity have been invoked as factors explaining relatively low rates of suicide among African Americans, despite the disproportionate presence of numerous socioeconomic risk factors for these outcomes in this community (Breslau et al., 2006).

## Exploring the relationship between religion and psychiatry

Despite the evidence for the salutary nature of religiosity among the general population and especially in minorities, analysis suggests that contemporary “mainstream” psychiatry still struggles in its efforts to integrate religion- or faith-based interventions (Galanter, 2005; Koenig, 2009). A systematic review of empirical papers published in four major psychiatric journals (over a 5-year period) found that only 1.2% of articles measured a religious variable (Weaver et al., 1998). Indeed, across disciplines, commentators have remarked on the predominantly areligious perspective of psychiatry, noting, for example, the complete lack of mention of religion in some core textbooks used in the education of mental health professionals (Galanter, 2005; McGrath, 2006). That said, there are signs of change in this domain. For example, the American Psychological Association has recently published a number of books exploring the meaningful integration of spirituality into therapeutic practice (e.g., Aten & Leach, 2008; Plante, 2009). Other new textbooks also include meaningful material on religion and mental health (e.g., Kay & Tasman, 2006). Still, the exploration of the impact of religiosity on mental health remains a minority pursuit and a somewhat neglected domain (in comparison, for example, with biological psychiatry), perpetuated in the main by social scientists rather than psychiatrists. Likewise, few faith-based interventions are developed, tested, or integrated into mainstream psychiatric services in western countries.

This situation could be a consequence of ongoing hostility (or indifference) to religion and religious worldviews within the institution of psychiatry (Allport, 1958; Bhugra, 1996), or within what Rose (1989) calls the “psy-disciplines.” This is an umbrella term used henceforth in this paper encompassing psychiatry, psychology, psychotherapy, and psychoanalysis. It has been argued that these attitudes derive from many sources, not least the historical aversion set by Freud to matters religious. Psy-professionals are much more likely to be atheists than both other health care professionals and the general population, with a recent study finding that psychiatrists are significantly less likely to be religious than other physicians (Curlin et al., 2007). An earlier seminal study indicated that 90–95% of the American general population are theists, in comparison to 30–40% of psy-professionals (Lukoff, Lu, & Turner, 1992). These authors conclude that “mental health professionals have tended to ignore or pathologize the religious and spiritual dimensions of life,” partly as a consequence of their own personal belief systems. Relatedly, it should be noted that mental health services in North America and Europe are often monocultural, monochrome, “White” institutions. Miranda, McGuire, Williams and Wang (2008) note that 93% of psychologists and 92% of social workers in the USA are White, whereas only 2% of psychologists and 4% of social workers are Black. Twelve percent of the U.S. population is Black/African American. Given that minorities are the prime bearers of religiosity, proreligious voices in mental health services may be sidelined within these services through the lack of diversity therein.

As stated, negative attitudes to religion in the psy-disciplines could be considered the residues of earlier orientations. It has been argued that these negative attitudes paradoxically became more entrenched as the psy-disciplines progressively rejected Freud as the 20th century advanced and the psy-disciplines embraced scientism as a principal ideological propeller and organizing worldview (Gould, 1996; Lewontin, Rose, & Kamin, 1984; Luhrmann, 2000; Mowrer, 1960; Rose, 1989). Considering “esoteric” matters such as religion may be considered inconsistent with the psy-disciplines’ quest to be taken seriously as “scientific” by emphasizing biological psychiatry, neuroscience, and genetic explanations. Distancing from religion may be deemed necessary to shore up their insecure identity as sciences.

Another factor explaining ambivalence towards the integration of religion in psychiatry concerns appropriate professional boundaries. Many psy-professionals may feel awkward discussing religious matters with patients given that this is a deeply personal domain, and may consider this a breach of boundaries, akin to discussing politics. Even professionals who are sympathetic to religion may fear being accused of proselytizing or moralizing if they devoted attention to religion in the clinical encounter. As well, mental health professionals may lack training which would help them overcome such fears (Galanter, 2005). That said, this situation may be changing in a more positive direction. Approximately 70% of U.S. medical schools now offer courses on spirituality and health, and some psychiatric residency programs include courses on spirituality, including learning how to take a “spiritual assessment” (Grabovac, Clark, & McKenna, 2008; Grabovac & Ganesan, 2003; Puchalski, 2006; Puchalski, Larson, & Lu, 2001). Again, it appears that bridges are being built between psychiatry and religion, which may ultimately be of benefit to patients, especially those from a minority background.

## Religious competence as cultural competence

Cultural competence has been defined as a set of skills and practices that lead to culturally appropriate services that respect patients’ ethno-cultural beliefs, values, attitudes, and conventions (Bhui, Warfa, Edonya, McKenzie, & Bhugra, 2007). Interest in cultural competence arose as ongoing research suggested large inequities in access and quality of psychiatric care between ethno-racial groups, with minorities seeking care less and dropping out more often (Vega, 2005; Whitley, 2007). Cultural competence was proposed as a concept and set of practices that could help reduce these disparities by making services and treatment options more responsive to ethno-cultural differences, and thus more attractive and engaging for minorities (Institute of Medicine, 2002; Surgeon General, 2001). Indeed, the seminal U.S. government New Freedom Commission on Mental Health (2003) posits that individual treatment should be appropriately tailored in light of ethno-cultural particularities. There is strong government commitment to the paradigm of cultural competence within the USA.

Cultural competence by definition includes religious competence, as individual religious orientation infuses patients’ beliefs, values, attitudes, and conventions. Not only does religion (or lack thereof) determine patients’ psychological and existential frameworks; it can also play a key role in determining behavioral variables (which, in turn, influence physiological variables) that have a direct bearing on mental health. For example, religiosity may influence use of alcohol, use of substances such as cannabis, patterns of sexual activity, sleep, and diet (the latter in the form of fasting often affecting medication adherence). Religious practices permeate most domains of life, and cannot be neatly compartmentalized or separated from everyday activities and concerns.

In any effort to understand patients’ religiosity, clinicians and agencies should be aware of Allport’s (1958) distinction between “interiorized” and “institutionalized” religion. Interiorized religion refers to beliefs and practices mostly conducted in private, for example devotional reading or private prayer. Institutionalized religion refers to beliefs and practices conducted in community, for example attending services or attending study groups of sacred texts. These are differentially experienced in diverse societies, with some groups being more community centered, and others where religious practice is mainly located in the home (Durkheim, 1912/2001). Moreover, individuals commonly shift their level and type of religious involvement and behavior across different social settings, contexts, and domains of life experience (Chaves, 2010).

Religious competence then requires clinicians and mental health services to be open, aware, and appropriately attentive to both the interiorized and institutionalized religious needs and orientations of patients, and to address these needs in a respectful, safe, and meaningful manner. Skilled clinicians can then mobilize patients’ religious orientations and resources to promote resilience, recovery, and healing.

In developing notions of religious competence, much can be learnt from efforts to shift the notion of cultural competence towards notions of “cultural safety” or “cultural humility” (Polaschek, 1998; Tervalon & Murray-García, 1998). These constructs posit that it must be a priority for clinicians and health care institutions to provide a safe place for discussion of cultural issues and identities, which will be received in a humble, respectful, and empathetic manner. This contrasts with technocratic notions of cultural competence which emphasize the learning of precise skills or competencies that can be used in dealing with patients from other ethno-cultural backgrounds (Whitley, 2007). Translating this to notions of religious humility or safety would involve creating a safe space (both interpersonally and at the level of the treatment provider) where religious matters can be discussed, addressed, and integrated into the treatment plan. This shift in stance is a first step towards implementing more precise practices and techniques of religious competence.

## Integrating religious competence: Challenges and recommendations

What does it mean for a practitioner or an institution to be religiously competent? As yet, there is no consensual answer to this question, but instead a lively debate about the kind of activities clinicians can (and should) engage in to elicit and integrate religious matters into patient care. It has been a consistent tenet of the biopsychosocial model that patient assessment must go beyond a simple symptom inventory. As such, an inquiry into spirituality and religion should become a routine aspect of assessment (Dein et al., 2010). Conducting this inquiry in a sensitive manner is an essential component of religious competence. Taking appropriate clinical action based on the elicited information is also a key part of religious competence. Fortunately, three prominent and overlapping templates are available to aid in such an enterprise.

Koenig (2008) argues that a psychiatrist can respectfully and successfully address a patient’s religious needs by conducting the following five activities: (a) taking a spiritual history; (b) respecting and supporting spiritual beliefs; (c) challenging beliefs; (d) praying with patients; and (e) appropriate consultation with clergy. Puchalski (2006; Puchalski & Romer, 2000) has developed a simple time-efficient yet effective tool named the FICA for making what she terms a “spiritual assessment.” The FICA inquires into the following four domains, through posing simple but relevant questions: (a) *Faith and belief*, “Do you have spiritual beliefs that help you cope with stress?”; (b) *Importance*, “What role do your beliefs have in regaining health?”; (c) *Community*, “Are you part of a religious or spiritual community? If so, is this of support to you and how?”; and (d) *Address in care*, “How would you like me as your healthcare provider to address these issues in care?”

Griffith and Griffith (2002) give additional advice and examples regarding how to talk to patients about their spiritual lives, and how to enhance the therapeutic alliance through a more extensive understanding of the patient’s religious views. They recommend that clinicians remain open to religion and spirituality by taking the “position of an anthropologist meeting another person from an unknown culture” (2002, p. 26), so that curiosity, openness, and wonder underpin the clinician’s approach. What this means in practice is close and sensitive listening to the patient’s narrative, with respect and openness to any religious or spiritual sensibilities, which they call “multi-channel listening.” They argue that this can best be achieved by asking probing but nonintrusive questions such as: “What sustains you through this illness?”; “What gives you hope when coping with this illness is most difficult?”; “Who truly understands what you are experiencing with this illness?”; and “How do you find comfort in your suffering?” They suggest that clinicians elicit and focus on information that can be used to promote “hard” outcomes such as medication adherence or specific health behaviors (e.g., substance use), as well as “soft” outcomes such as enhancing hope and agency. Interventions might include encouraging and facilitating greater participation in a religious community, engagement in rituals or spiritual practices, or consultation of scripture. It also means helping the patient confront or challenge religious or spiritual beliefs and practices that may be destructive to mental health, for example a belief that mental illness is a punishment from God (Griffith, 2010).

All three of these approaches emphasize the importance of asking short but specific questions to elicit patients’ religious and spiritual beliefs, behaviors, and attitudes. Questions should be posed in a nonjudgmental and sensitive manner to ensure a sense of safety in the clinical encounter. In doing so, patients must be free to disengage or change topic, and clinicians should also drop the topic if there is a clear lack of interest. There should be no coercion to discuss religion in the interview, which of course, is a general principle of medical ethics (Beauchamp & Childress, 2001), but which has particular significance in this domain since some patients may have had negative experience associated with religion, for example, survivors of clerical sexual abuse (Griffith, 2010). An empathic and sensitive approach to interviewing will also allow appropriate responses to those holding minority or committed nonreligious worldviews such as atheism, a group neglected in mental health research and practice (Whitley, 2010).

In acting on the information elicited, a sensitive clinician can integrate patients’ religiosity into the treatment plan. For example, the clinician may encourage the patient to engage in regular prayer or consultation of sacred texts, if this appears to be a source of strength and support. The moral codes associated with a patient’s religious worldview might influence behavior and decision making (e.g., religious injunctions to abstain from the abuse of alcohol). These can be utilized by the clinician to help meet clinical goals, for example, by recommending that a religiously inclined patient with substance use disorder join Alcoholics Anonymous. The clinician may also encourage patients to utilize communal and social networks associated with their religious congregation to garner social, emotional, and instrumental support to aid recovery. Clinicians may keep a list of religious organizations and congregations which have demonstrated that they are especially open to and supportive of people living with mental illness.

Indeed, all three approaches described above emphasize the importance of community and clergy in healing. Clergy and/or chaplains can be integrated in various ways. Firstly, religiously inclined patients with more mild and moderate psychiatric disorders may be “triaged” to the appropriate clergy, chaplains, or pastoral counselors for psychosocial support. While clergy should not be considered a replacement for appropriate psychiatric care, they may be able to provide invaluable assistance in areas such as uplifting morale or enhancing community reintegration. Clergy also can be used as “culture brokers” in difficult cases. In this role, they can mediate between a patient and a clinical team, in times where the patient’s religiosity or religious background appears to be significantly influencing pathogenesis, pathoplasticity, patterns of service utilization, or explanatory models.

Effective communication between clergy and clinicians must be reciprocal, in order to build capacity in mental health as well as reduce stigma. Research indicates that clergy are the first port of call for many people (particularly in some ethno-cultural minority groups) during early or prodromal stages of mental illness (Ellis et al., 2010; Koenig, 1998). However, depending on circumstance, clergy may have little or no training in how to recognize or deal with people in these early stages. Clinicians can help educate clergy about mental illness, in order to prepare them to better identify and refer people with mental illness to appropriate services (Leavey, 2008). Clergy can also play a key role in reduction of stigma, using their position of prominence within their congregation (and the wider community) to emphasize the common humanity of people living with mental illness. Clergy may also assist addressing complex issues that arise in the hospital care of people, particularly those with strong religious identities and involvement (Popovsky, 2010).

## Implications for training and clinical practice

In order to meet the previously mentioned recommendations, adequate training must be provided for clinicians, both during undergraduate and postgraduate training as well as through continuing professional development. As previously stated, more than 70% of medical schools now teach courses on spirituality and health, with some specific courses in psychiatric residency programs (Puchalski et al., 2001). This is promising and further sessions and seminars can be built onto these in order to better impart religious competence in psychiatry. This should deal with the previously discussed material on inquiring into religious orientation. It might also involve training in how to respond to common challenges faced by clinicians when dealing with a patient for whom religion is of ontological importance. Though many people find solace and support from religion, others will find elements of their religion burdensome. For example some may hold a punitive view of God, seeing their illness as a punishment for sin; they may fear hell as an ultimate destiny because of their perceived transgressions (Griffith, 2010). Some patients may have a preoccupation with eschatological texts such as the *Book of Daniel* or the *Book of Revelation*. Others may feel emotions such as guilt, shame, abandonment, and despair, consequent upon their religiosity. Role-playing and training in these issues may help clinicians when dealing with these challenges in the real world.

One other area where more efforts could be made is general openness to language and discourse which deviates from the received secular wisdom of the psy-disciplines. Again this can be best imparted through systematic training. For example, many people with mental illness use overtly moral and theological discourse to describe events and happenings in their lives (Whitley, Kirmayer, & Groleau, 2006). This contrasts with the language common in the psy-disciplines, which typically discourage use of moral and theological language, preferring to frame happenings in the amoral language of clinical psychology or neuroscience (Kleinman, 2006; Mowrer, 1960; Rose, 1989).

Acknowledgement, if not integration of theological language and concepts into service provision may make services much more attractive to theistically inclined patients. This does not mean that religious language is accepted uncritically. As Koenig (2008) notes, clinicians should challenge spiritual beliefs where necessary. However, this should be done in a safe and respectful manner, rather than in a dismissive or paternalistic one.

Religious competence consists of more than optimizing the clinical interaction. It also involves reorientations at the level of mental health services. Mental health services can weave religion into their core activities, or partner with religious bodies to engage and retain patients. This could involve making chaplains readily available to support patients. It could also involve the formation of spiritual reading groups, or peer prayer groups, or celebrating key events in the liturgical calendar as forms of recognition. Indeed, clinics and staff that are religiously competent may be better placed to engage people with mental illness, and enhance their recovery processes. Throughout history organizations which have actively attempted to integrate religion/spirituality and psychiatry have reported much success. A well-documented case is Hope Haven in Iowa (Bussema & Bussema, 2000, 2007). Such enterprises provide encouraging examples to others wishing to become more religiously competent.

Indeed, community mental health services may learn something from popular and more informal addictions services such as Alcoholics Anonymous. These have incorporated religion and spirituality to a much greater extent than routine mental health services, with much evidence suggesting that these services are more popular among people with substance use disorder (and dual diagnosis) and even more effective than routine services (Aase, Jason, & Robinson, 2008; Booth & Martin, 1998; Galanter, 2006).

To realize the values of patient-centered care (Berwick, 2009), mental health clinicians and institutions need to make concrete efforts to improve religious competence at both micro (interpersonal) and meso (organizational) levels. Harnessing the individual’s moral resources and exploiting their social ecology in order to facilitate progress is a key aspect of recovery (Anthony, 1993; Deegan, 1996, 1997). As such, sensitivity to religion can be meaningfully incorporated into services and treatment in settings where large numbers of the clientele are religiously inclined.

Of course, such efforts may be perceived by some as violating fundamental tenets of secularism that have become explicit values in many societies (Taylor, 2007). Attention to religion may also lay mental health services open to attack as endorsing outmoded or backward worldviews, and may disturb those in the mental health system who have welcomed the distancing from religion that the psy-disciplines have afforded. Somewhat paradoxically, in an effort to create an inclusive public sphere, some societies have worked to exclude expressions of religion in public institutions (Berger, 1999). As such, individual-level, discipline-level, provider-level, and societal-level factors may all interact to prevent the optimal integration of religious competence in psychiatry.

## Conclusion

In this paper I argue that religiosity is often insufficiently recognized, explored and harnessed by clinicians when treating people with mental illness, especially those from ethno-cultural minorities. Clinicians and mental health services must take religion seriously as a resource to enhance recovery. Religious competence is not at all incompatible with belief in the efficacy of conventional mental health services. Religion and psychiatry can be employed in mutually reinforcing ways to enhance recovery and rehabilitation. Indeed, some writers have interpreted past and present experience of mental illness as a positive transforming experience that builds perseverance, character, and hope—virtues which are often mentioned as synonymous with recovery from mental illness (Anthony, 1993; Deegan, 1996, 1997). These virtues are also encouraged by almost all of the major world religions.

The wider point is that any mental health clinic that claims to be recovery-centered needs to assess the culture of the community it serves, and work with the moral resources and social ecology of that culture to facilitate recovery. It may be relatively easy for a mental health service to provide bilingual services in Spanish and English as part of being a “culturally competent” organization. However, I have argued that the history, culture, and political climate in 21st-century secular democracies work in concert with the long-standing ambivalence towards religion within mainstream psychiatry to constrain the development and integration of “religious competence” into publically funded mental health services. This ambivalence is contrary to the mental health discipline’s provenance and tradition within the humanities and theology, as well as science. In this era of patient-centered care and recovery-oriented practice, it is hoped that religious competence will become an important component of psychiatric practice.
